# Erythropoietin does not activate erythropoietin receptor signaling or lipolytic pathways in human subcutaneous white adipose tissue in vivo

**DOI:** 10.1186/s12944-016-0327-z

**Published:** 2016-09-17

**Authors:** Britt Christensen, Birgitte Nellemann, Jens Otto L. Jørgensen, Steen B. Pedersen, Niels Jessen

**Affiliations:** 1 Department of Endocrinology and Internal Medicine, NBG/THG, Aarhus University Hospital, Nørrebrogade 44, 8000 Aarhus C, Denmark; 2 Research Laboratory for Biochemical Pathology, Institute for Clinical Medicine, Aarhus University Hospital, Aarhus, Denmark; 3 Department of Molecular Medicine, Aarhus University Hospital, Aarhus, Denmark

**Keywords:** rHuEpo, Darbepoietin-α, Mitochondria, Lipolysis

## Abstract

**Background:**

Erythropoietin (Epo) exerts direct effects on white adipose tissue (WAT) in mice in addition to its erythropoietic effects, and in humans Epo increases resting energy expenditure and affect serum lipid levels, but direct effects of Epo in human WAT have not been documented. We therefore investigated the effects of acute and prolonged Epo exposure on human WAT in vivo.

**Method:**

Data were obtained from two clinical trials: 1) acute Epo exposure (rHuEpo, 400 IU/kg) followed by WAT biopsies after 1 h and 2) 10 weeks treatment with the erythropoiesis-stimulating agent (ESA) Darbepoietin-alpha. Biopsies were analyzed by PCR for Epo receptor (Epo-R) mRNA. A new and highly specific antibody (A82, Amgen) was used to evaluate the presence of Epo-R by western blot analysis in addition to Epo-R signaling proteins (Akt, STAT5, p70s6k, LYN, and p38MAPK), activation of lipolytic pathways (ATGL, HSL, CGI-58, G0S2, Perilipin, Cidea, Cidec, AMPK, and ACC), and mitochondrial biogenesis (VDAC, HSP90, PDH, and SDHA).

**Results:**

No evidence of in vivo activation of the Epo-R in WAT could be documented despite detectable levels of Epo-R mRNA.

**Conclusion:**

Thus, in contradiction to animal studies, Epo treatment within a physiological relevant range in humans does not exert direct effects in a subcutaneous WAT.

## Background

Erythropoietin (Epo), a glycoprotein produced in the kidneys and known for its erythropoietic effects, is used to treat nephrogenic anemia. Moreover, Epo receptors (Epo-R) of alleged functionality have been identified in numerous tissues [1]. In particular, data in mice suggest high expression of Epo-R mRNA in spleen and in white adipose tissue (WAT) [2].

Studies in end-stage renal disease (ERSD) patients show that serum levels of triglycerides, total cholesterol, and LDL decrease to normal levels after Epo treatment [3–5], but acute administration of rHuEpo in healthy subjects do not significantly affect lipid levels, although a tendency to increased free fatty acid (FFA) levels have been observed [6]. However, prolonged treatment to healthy humans with an erythropoiesis-stimulating agent (ESA) significantly increases serum FFA levels and hepatic lipid content [7].

In mice overexpressing the Epo gene, increased fat oxidation and an up-regulation of genes involved in lipid metabolism in skeletal muscle has been shown [8]. On the other hand, mice with a lack of Epo-R, except for the bone marrow, have a significantly higher body weight, increased fat mass and increased serum TG levels [2, 9]. Furthermore, a reduction in body weight due to reduced WAT mass in obese mice is found after Epo treatment [2, 8–11].

The mechanism by which Epo regulates fat metabolism and body weight is currently unknown. One explanation could be an increase in resting energy expenditure including fat oxidation [2], but only the former has been documented in healthy human subjects [6, 7]. As regards direct peripheral actions of Epo, there is no evidence to support such effects in human skeletal muscle [12, 13]. The effect of Epo treatment on WAT in humans has so far not been evaluated, but Epo-R protein and mRNA has been detected in WAT from mice in addition to a suppressive effect of Epo on fat accumulation and preadipocyte differentiation and expansion in vitro [2, 14]. Epo administration in mice is also associated with induction of brown adipose tissue (BAT) like features in WAT with increased mitochondrial content and uncoupling [9]. Furthermore, adipocyte specific deletion of the Epo-R in mice lead to obesity and decreased insulin sensitivity [9]. In contradiction, specific knockdown of the adipocyte Epo-R showed that Epo signaling at physiological levels was not essential for WAT metabolism [15].

The aim of the current study was to evaluate both the acute and prolonged effects of Epo treatment on human subcutaneous WAT on; 1) Epo-R expression, 2) activation of Epo-R related signaling pathways, and 3) direct and indirect effects on lipolysis and WAT metabolism.

## Methods

### Subjects

#### Acute study

Ten healthy young men (23 (20–28) years, 179 (173–192) cm, and 77 (68–90) kg, median (range)) participated after providing written informed consent, in adherence to the declaration of Helsinki. The Local Ethical Committee of Central Denmark Region (M-2008-0016) approved the study and it was further registered at clinicaltrials.gov (M-20080035). Results regarding whole body and skeletal muscle metabolism from the same study have previously been published [6].

#### Prolonged study

Eighteen healthy untrained men (placebo group: 23 (21–35) years, 183 (172–191) cm, and 80.1 (67.7–96.5) kg; ESA group: 22 (19–29) years, 186 (177–198) cm, and 80.3 (70.7–102.5) kg, median (range)) were included after receiving oral and written information and written informed consent to participate, in accordance with the declaration of Helsinki. This study was approved by the Local Ethical Committee of Central Denmark Region (M-20110035) and registered at clinical trials (NTC01320449). This study is part of a larger study where the effects of erythropoietin and endurance training on skeletal muscle and whole body metabolism have been compared [7, 13, 16, 17].

### Study design

#### Acute study

The subjects were in a single-blind, crossover design randomized to receive placebo and rHuEpo on two occasions with a 14-day wash-out period in-between. On the experimental days, the subjects arrived fasting (from 10 pm the evening before, water allowed) at the clinical research unit in the morning. Placebo (saline) or rHuEpo (Epoietin alpha, Eprex, 400 IU/kg) were administered i.v. and subcutaneous fat biopsies were collected 1 h post administration from the abdomen by liposuction under local anesthesia. Fat biopsies were cleaned from blood, frozen in liquid nitrogen, and stored at −80 °C until further analysis. The experiments were performed under thermo neutral conditions (21–23 °C).

#### Prolonged study

Only subjects fulfilling the following criteria were included in the study: maximal oxygen uptake (VO_2_max) below 50 ml/min/kg, age between 18 and 35, body mass index (BMI) between 18 and 29 kg/m^2^, normal blood pressure <135/85, and a hematocrit <45 %. The subjects were randomly assigned to either a placebo group (saline, *n* = 9) or an Epo group (*n* = 9). The subjects were in a single blinded manner treated with either an erythropoiesis-stimulating agent (ESA) (Darbepoietin-α, Aranesp, Amgen, Thousand Oaks, CA, USA) or placebo (saline) for 10 weeks. ESA/placebo were administrated subcutaneously once weekly and the ESA dose was 40 μg for the first 3 weeks and 20 μg for the remaining 7 weeks as previously detailed [7]. Subcutaneous fat biopsies were collected from the abdomen with liposuction under local anesthesia, before and after the 10 weeks treatment. The biopsies were cleaned for blood, frozen in liquid nitrogen, and stored at −80 °C until further analysis. The procedure was performed under thermo neutral conditions (21–23 °C). The participants were instructed to refrain from hard physical activity, alcohol intake, or dietary changes 3 days prior to collection of the biopsies. On the day of biopsy collection the subjects arrived at the clinical research unit after an overnight fast (water intake allowed). During the study period the participants were informed not to change their level of physical activity or dietary habits.

### Epo-R mRNA analysis

Subcutaneous WAT from the subjects in the acute study (placebo treatment), bone marrow, and K-562 cells were used for RNA extraction using TRIzol (Gibco BRL, Life Technologies, Roskilde, Denmark) and homogenized with 1 tungsten bead (Qiagen, Germantown, MD, USA) using a Mixer Mill. RNA was quantified by measuring absorbance at 260 and 280 nm using a NanoDrop 8000 Spectrophotometer (Thermo Scientific Pierce, Waltham, ME, USA). Integrity of the RNA was checked by visual inspection of the 2 ribosomal RNAs on an agarose gel.

cDNA was synthesized using a Verso cDNA kit (cat# Ab-1453, Thermo Fischer Scientific, Waltham, MA, USA) with random hexamer primers. The levels of Epo receptor mRNA was investigated, b2microglobolin were used as housekeeping gene. For Epo receptor PCR a RealTime ready Assay (Roche Diagnostics Corporation, 9115 Hague Road, Indianapolis, IN 46250-0414 USA) was used. In this assay the following primers were used: Epo-R: 5′-GCATTGCTGATTTTGTCTGC-3′ and 5′-AAATACTGCAAGGTTGTGGTTTC-3′ together with a small Universal hydrolysis probe substituted with Locked Nucleic Acids from Roche (no152). No amplification was observed in negative control tubes (without reverse transcription or with only water). The b2microglobulin PCR was performed as a SYBR-Green assay using KAPA SYBR® FAST qPCR Kit (Kapa Biosystems, Inc. Woburn, MA, USA) with primers: 5′-GAGGCTATCCAGCGTACTCC-3′ and 5′-AATGTCGGATGGATGAAACCC-3′.

The PCR-reactions were performed in duplicate in a LightCycler 480 (Roche Applied Science) using the following protocol: EPO-receptor assay: One step at 95 °C for 3 min., then 95 °C for 10 s., 60 °C for 30 s., and 72 °C for 11 s. b2microglobulin assay: One step at 95 °C for 3 min., then 95 °C for 10 s., 60 °C for 20 s., and 72 °C for 10 s, and finally a melting curve analysis was performed (for the b2microglobulin assay). The increase in fluorescence was measured in real time during the extension step. The relative gene expression was estimated using the default “Advanced Relative Quantification” mode of the software version LCS 480 1.5.0.39 (Roche Applied Science).

### Western blot analysis

Approximately 100 mg subcutaneous fat tissue was homogenized in homogenization buffer (*Acute study:* 20 mM HEPES, 10 mM NaF, 1 mM Na_3_VO_4_, 1 mM EDTA, 5 % SDS, 50 μg/ml Soybean trypsin inhibitor, 4 μg/ml Leupepsin, 0.1 mM Benzamidine, 2 μg/ml Antipain, and 1 μg/ml Pepstatin; *Prolonged study:* 50 mM HEPES, 20 mM NaF, 2 mM Na_3_VO_4_, 5 mM EDTA, 5 % SDS, HALT, 5 mM NAM, 10 μM TSA) on a Precellys 24 (Bertin technologies, Montigny-le-Bretonneux, France). Hereafter, samples were thermo mixed at 37 °C and 500-1000 rpm for 1 h, followed by centrifugation at 14,000 x g for 20 min at room temperature. The homogenate was carefully separated from the lipid layer by a syringe, snap frozen, and centrifuged again, in order to purify the homogenate even further. The homogenate was frozen in liquid nitrogen and stored at -80 °C until further analysis.

In short, western blotting was performed as follows; 10 μl homogenate was loaded onto a 4–15 % SDS gel (Criterion TGX stain-free gels, Bio-Rad, Hercules, CA, USA), followed by electro blotting onto a PVDF membrane. The stain-free technology was used to ensure equal loading [18]. Membranes were blocked with 2.5 % skimmed milk for 2 h before the primary antibody was added and incubated overnight at 4 °C. The following primary antibodies were used: From Cell signaling, Danvers, MA, USA; phospho-LYN (Thr507) (#2731), LYN (#2732), phospho-Akt (Ser473) (#9271), phospho-Akt (Thr308) (#9275), pan-Akt (#4691), phospho-p70S6k (Thr389) (#9205), p70S6k (#9202), phospho-STAT5 (Thr694) (#9359), STAT5 (#9358), phospho-p38MAPK (Thr180/Thr182) (#9211), p38MAPK (#9212), phospho-HSL (Ser660, corresponding to Ser650 in humans) (#4126), phospho-HSL (Ser563, corresponding to Ser552 in humans) (#4139), phospho-HSL (Ser565, corresponding to Ser554 in humans) (#4137), HSL (#4107), ATGL (#2138), HSP60 (#12165), SDHA (#11998), PDH (#3205), VDAC (#4661), phosphor-AMPKα (Thr172) (#2531), and PKA (#9624), from Abcam, Cambridge, UK; CGI-58 (#ab183739), anti-β-actin (#ab8227), and G0S2 (#ab80353), from Novus bio, Littleton, CO, USA; Cidea (#NB100-94219), from Abnova, Atlanta, GA, USA; Cidec (#H00063924-M07), from Millipore, Darmstadt, Germany; AMPKα pan (#07-181) and phospho-ACC (Ser79) (#07-303), from Amgen, Thousand Oaks, CA, USA; anti-Epo-R (#A82), from Southernbiotech, Birmingham, AL, USA: HRP streptavidin (#7100-05), from Santa Cruz, Dallas, TX, USA; G0S2 (#sc-133424), and from Pierce antibody production, Thermo scientific, Waltham, MA, USA; Perilipin (#PA1-1052). Following several washes, the membrane was incubated with the secondary antibody (donkey-anti-rabbit IgG, #NA934, Amersham, GE Healthcare, Pittsburgh, PA, USA/goat-anti-rabbit IgG, #sc-2054, Santa Cruz, Dallas, TX, USA) for 1 h at room temperature. Proteins were visualized by chemiluminescence detection system (Super signal dura extended duration substrate, Pierce, Thermo Scientific, Waltham, MA, USA/Clarity Western ECL substrate, Bio-Rad, Hercules, CA, USA #170-2054) using a ChemiDoc^TM^ MP imaging system (BioRad, Hercules, CA, USA). Precision Plus Protein All Blue Prestained Protein Standard (BioRad, Hercules, CA, USA #1610373) was used as molecular weight marker.

### Haematoxylin/Eosin staining

To evaluate adipocyte morphology, selected WAT biopsies from the prolonged study was fixed in cold (4 °C) 4 % formaldehyde (pH 7.0) for 2 days and embedded in paraffin, after which sections of 3 μm were obtained. After de-waxing and rehydration, the sections were stained with Haematoxylin and Eosin and examined under an Olympus light microscope (Olympus BX50).

### Statistics

Due to a low sample size and non-normally distributed data, a Wilcoxon signed-rank test was used to test for treatment effect on intracellular signaling in the acute study. Results are shown as median and 25 % and 75 % percentiles. A two-way ANOVA was used to analyze results from the prolonged study, QQ-plots and plots of residuals vs. the fitted values checked normality, and data were log-transformed when not normally distributed. The level of significance was set to *p* < 0.05. Results are presented as means ± SE. Statistical analyses were made in STATA version 12 (StataCorp, Collage Station, TX, USA) and graphical presentations were made in Sigmaplot version 11.0 (Systat Software, San Jose, CA, USA).

## Results

### Epo-R mRNA and protein in subcutaneous WAT

**Fig. 1:**
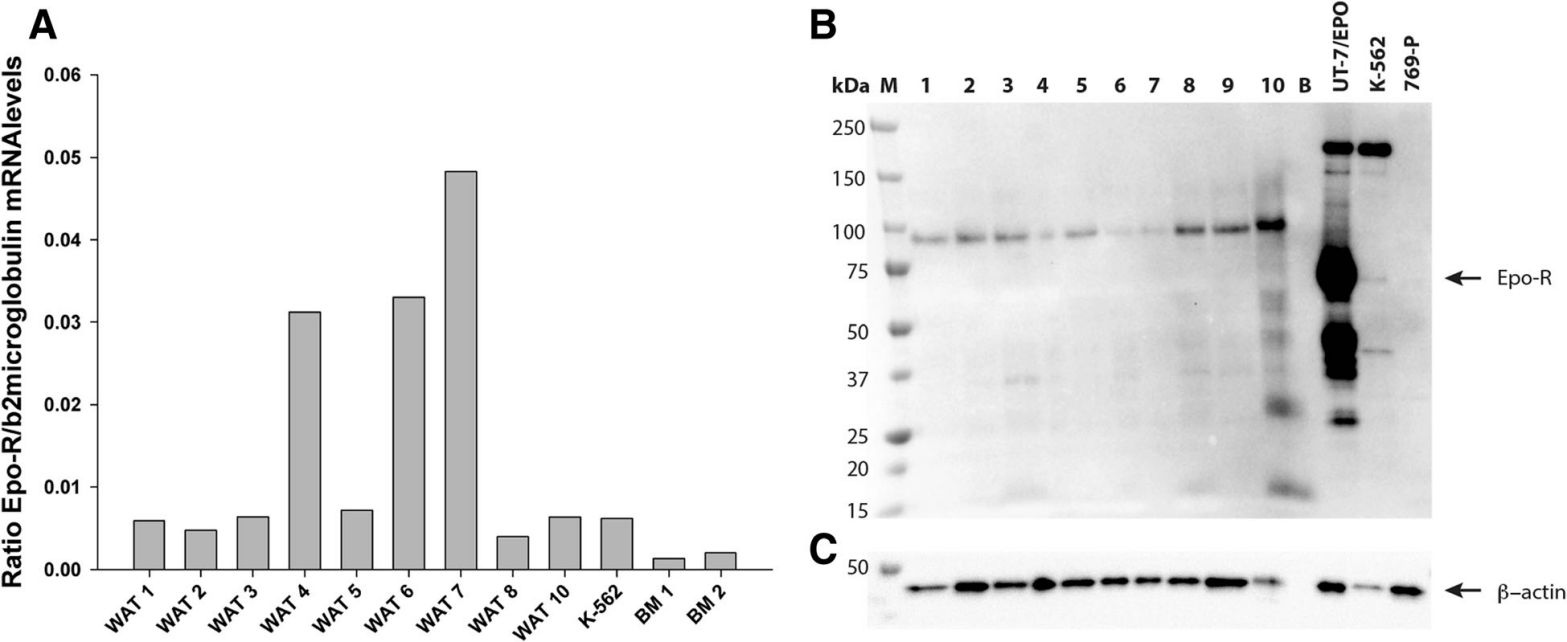
Epo-R protein in subcutaneous fat tissue. Epo-R gene expression (**a**) in human WAT from the acute study in the placebo situation. b2microglobolin was used as housekeeping gene control. Western blot analysis against the Epo-R (**b**) and β-actin (**c**) in human abdominal subcutaneous fat tissue. Positive controls; K-562 (Abcam) and UT-7/Epo cells (Amgen), negative control: 769-P cells (Amgen), bone marrow; BM, blank; B, and marker; M. The expected size of the Epo-R and β-actin are indicated by an arrow

Epo-R mRNA was detected in WAT from all subjects (average ct level of 29.0 cycles), as well as in the positive K-562 cells (ct level of 29.6 cycles). A slightly lower level of Epo-R mRNA was observed in bone marrow (average ct level of 29.3 cycles) (Fig. 1a).

No band corresponding to the Epo-R (59kD) was identified in any of the fat biopsies or the negative control 769-P cells (Amgen, Thousand Oaks, CA, USA), however a band was identified in the two positive controls, K-562 (Abcam, Cambridge, UK) and UT-7/Epo cells (Amgen, Thousand Oaks, CA, USA) (Fig. 1b + c).

### Epo-R signaling in subcutaneous WAT

**Fig. 2:**
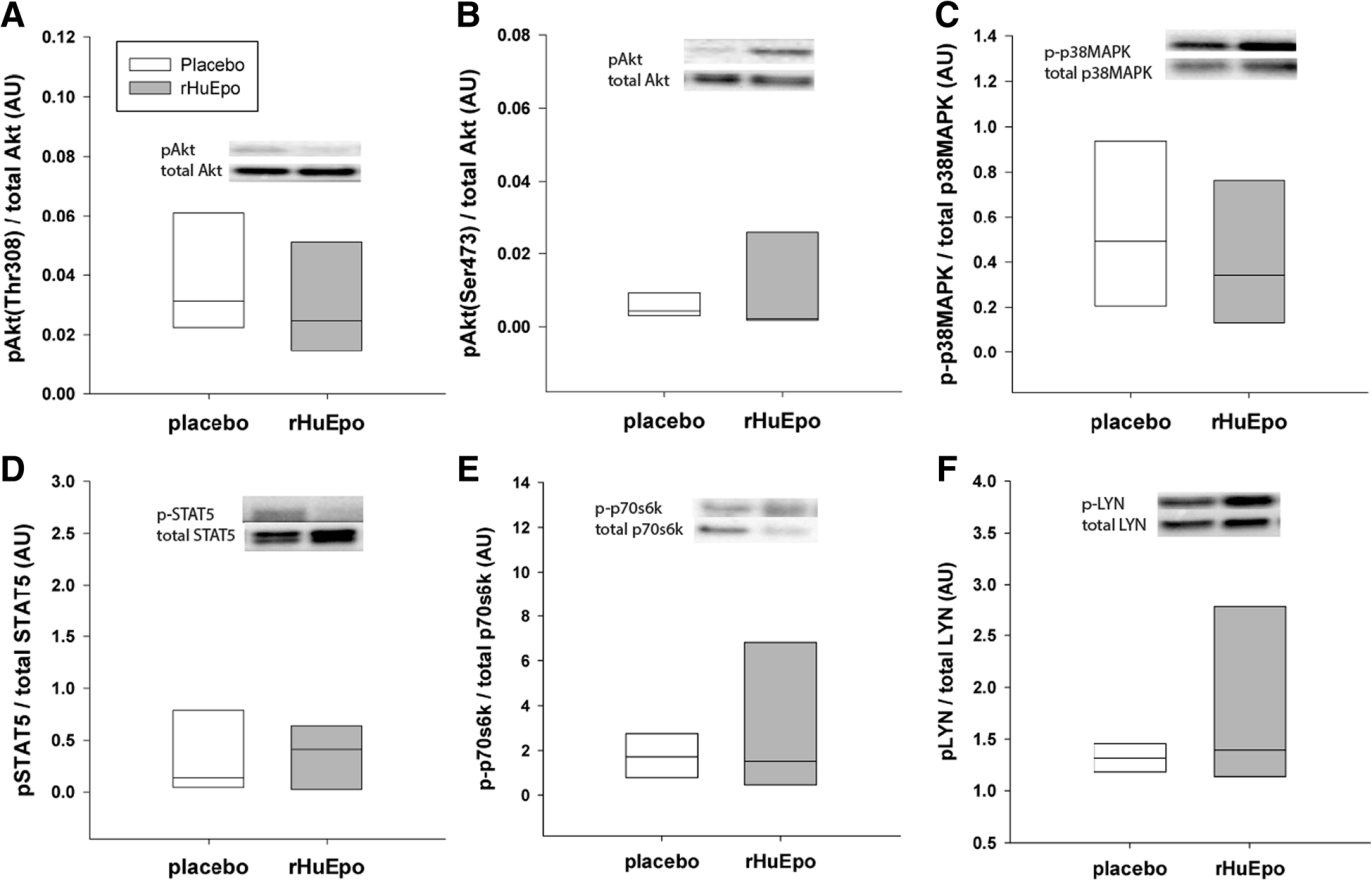
Epo-R signaling after acute rHuEpo administration. Western blot analysis for activation of relevant signaling proteins downstream of the Epo-R. Phosphorylation levels are measured 1 h post placebo/rHuEpo treatment, and normalized to the total levels of the given protein. The median and 25 % and 75 % percentiles are shown for placebo (*white bar*) and rHuEpo treatment (*gray bars*). Two subjects were omitted from the analysis due to inadequate homogenization, thus, *n* = 8. No statistical significant differences in phosphorylation levels were detected between treatments using a Wilcoxon signed-rank test

Western blot analysis was used to measure phosphorylation levels of pertinent proteins indicative of Epo-R activation and signaling. Phosphorylation levels were measured 1 h post placebo/rHuEpo treatment. Neither p-Akt (Thr308, *p* = 0.915; Ser473, *p* = 0.244), p-STAT5 (*p* = 0.309), p-p70s6k (*p* = 0.405), p-LYN (*p* = 0.245), or p-p38MAPK (*p* = 0.735) levels were significantly different between placebo and rHuEpo treatment (Fig. 2).

### Alterations in lipolysis after acute rHuEpo treatment

**Fig. 3:**
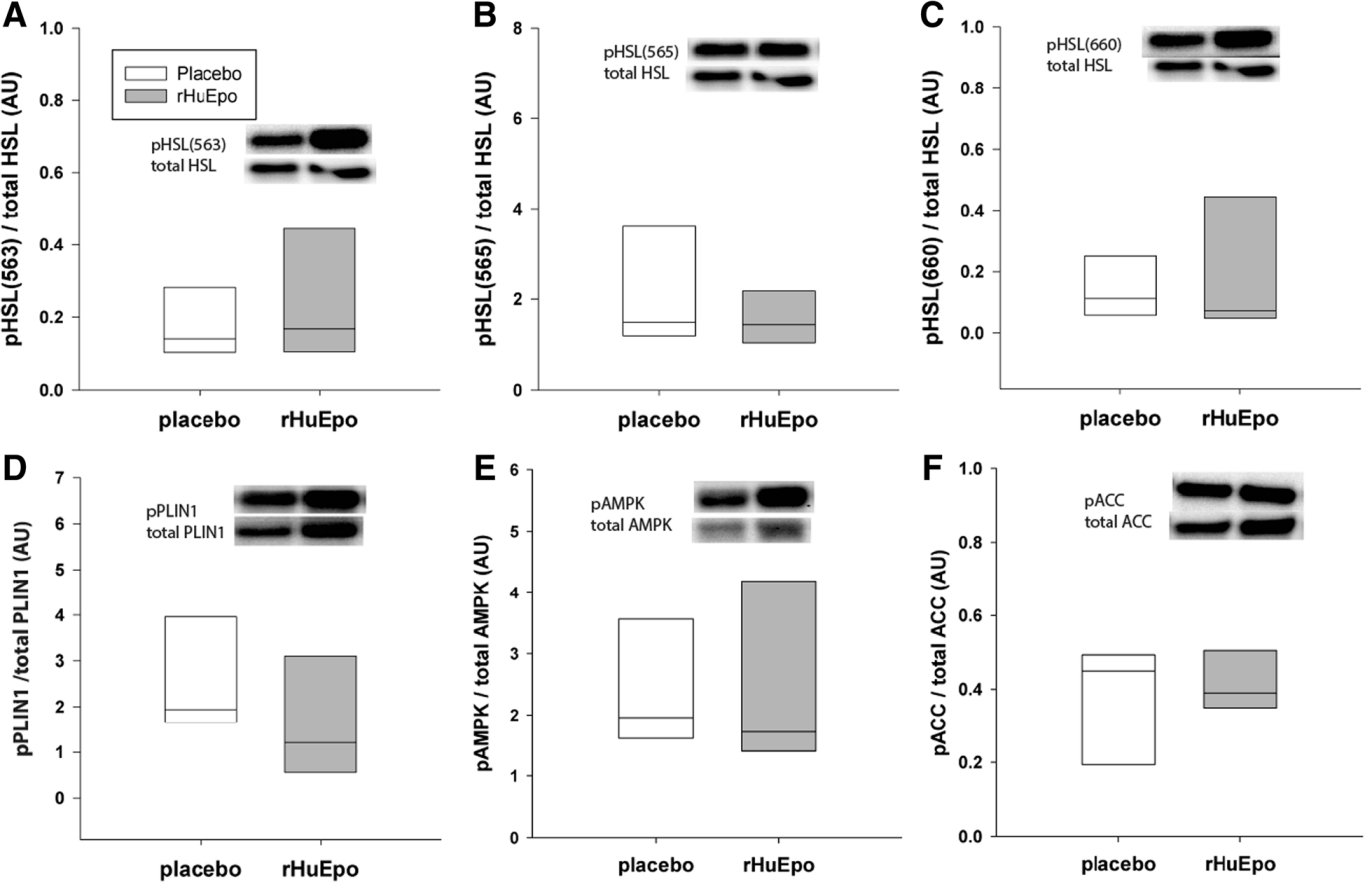
Lipolytic pathways after acute rHuEpo administration. Western blot analysis evaluating activation of different proteins involved in lipolysis. Phosphorylation levels are measured 1 h post placebo/rHuEpo treatment, and normalized to the total levels of the given protein. The median and 25 % and 75 % percentiles are shown for placebo (*white bar*) and rHuEpo treatment (*gray bars*). Two subjects were omitted from the analysis due to inadequate homogenization, and 1–2 subjects because of shortage of homogenate, thus, *n* = 6–7. No statistical significant differences in phosphorylation levels or total protein levels were detected between treatments using a Wilcoxon signed-rank test

Acute rHuEpo administration did not induce any differences in the phosphorylation levels of p-HSL (Ser563, *p* = 0.259; Ser565, *p* = 0.892; Ser660, *p* = 0.480), p-ACC (*p* = 0.132), p-Perilipin (*p* = 0.295), or p-AMPK (*p* = 0.665). Furthermore, total protein levels of G0S2 (*p* = 0.276) did not change (Fig. 3).

### Alterations in lipolysis and markers of metabolism after prolonged ESA treatment

**Fig. 4:**
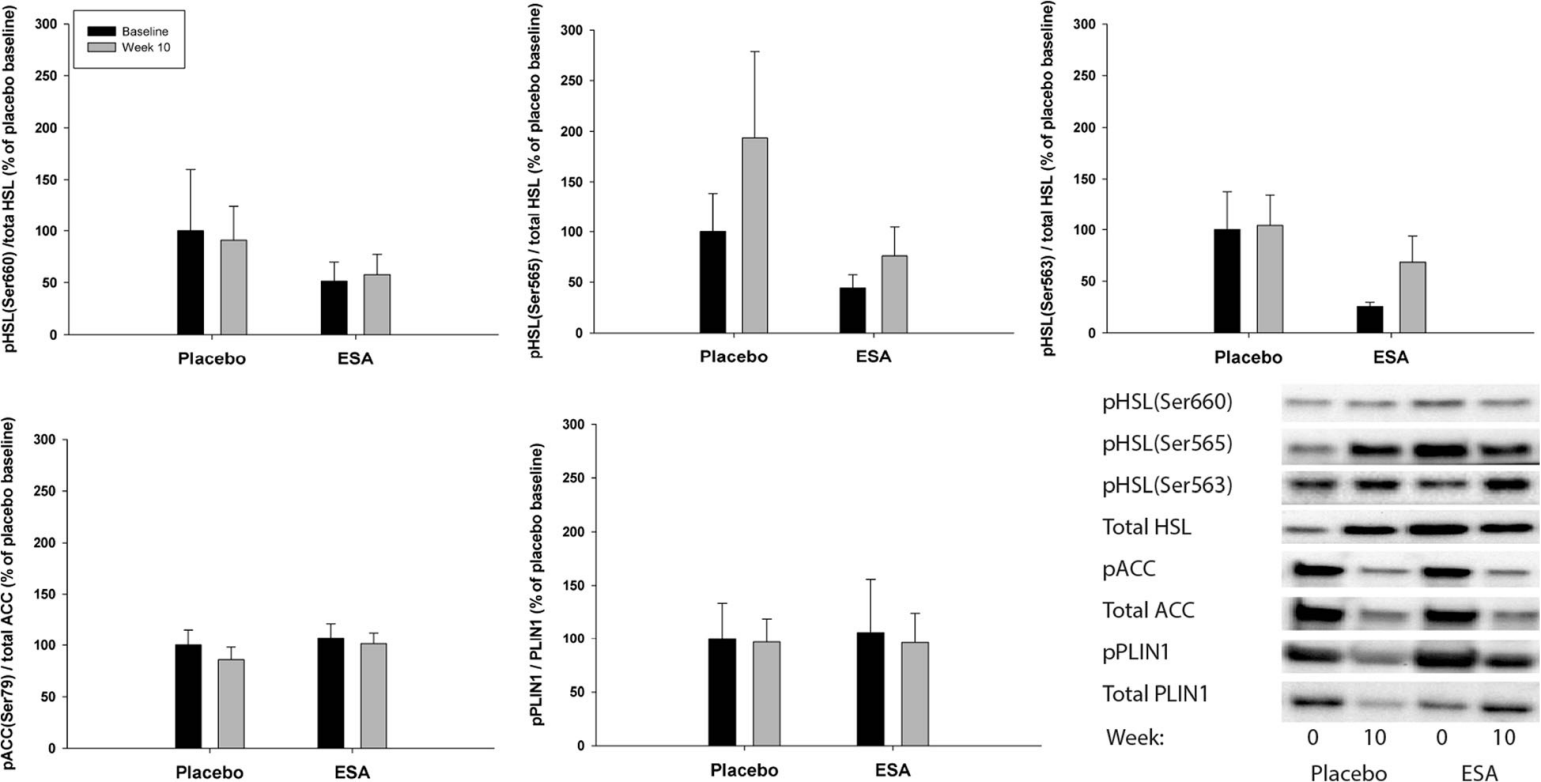
Phosphorylation levels of pertinent proteins involved in lipolysis after prolonged ESA treatment. Western blot analysis of phosphorylation levels of pertinent proteins related to lipolysis in adipose tissue. Biopsies were collected before (*black bars*) and after 10 weeks (*gray bars*) of treatment with either placebo or ESA. Phosphorylation levels were normalized to the total protein level for the given protein. Furthermore, the relative increases were measured and the placebo-baseline measurements were set to average 100 %. No effect of ESA treatment or time was found for any of the measured phosphorylation levels (*n* = 9)

**Fig. 5:**
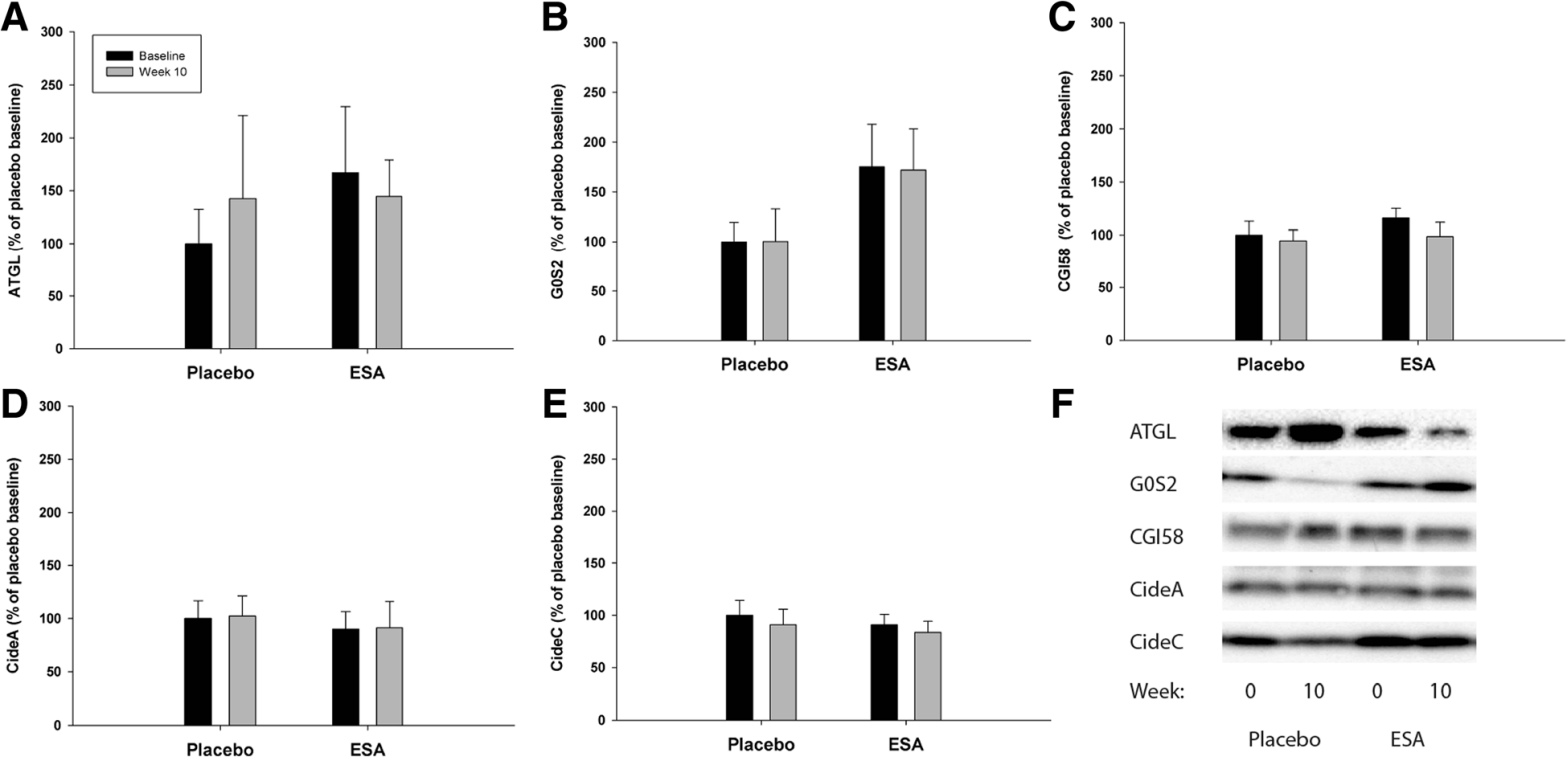
Total protein levels of proteins involved in lipolysis after prolonged ESA treatment. Western blot analysis of pertinent proteins related to lipolysis in adipose tissue. Biopsies were collected before (*black bars*) and after 10 weeks (*gray bars*) of treatment with either placebo or ESA. Data were normalized to total protein level in the gel. Furthermore, the relative increases were measured and the placebo-baseline measurements were set to average 100 %. No effect of ESA treatment or time was found for any of the measured proteins (*n* = 9)

Changes in both phosphorylation (Fig. 4) and total protein levels (Fig. 5) of proteins involved in lipolysis in adipose tissue after prolonged treatment with ESA were evaluated. We did not record changes in any of the proteins analyzed (two-way ANOVA, interactions *p* > 0.05, time effects *p* > 0.05, ESA effects *p* > 0.05).

### Haematoxylin/Eosin staining of WAT after prolonged ESA treatment

**Fig. 6:**
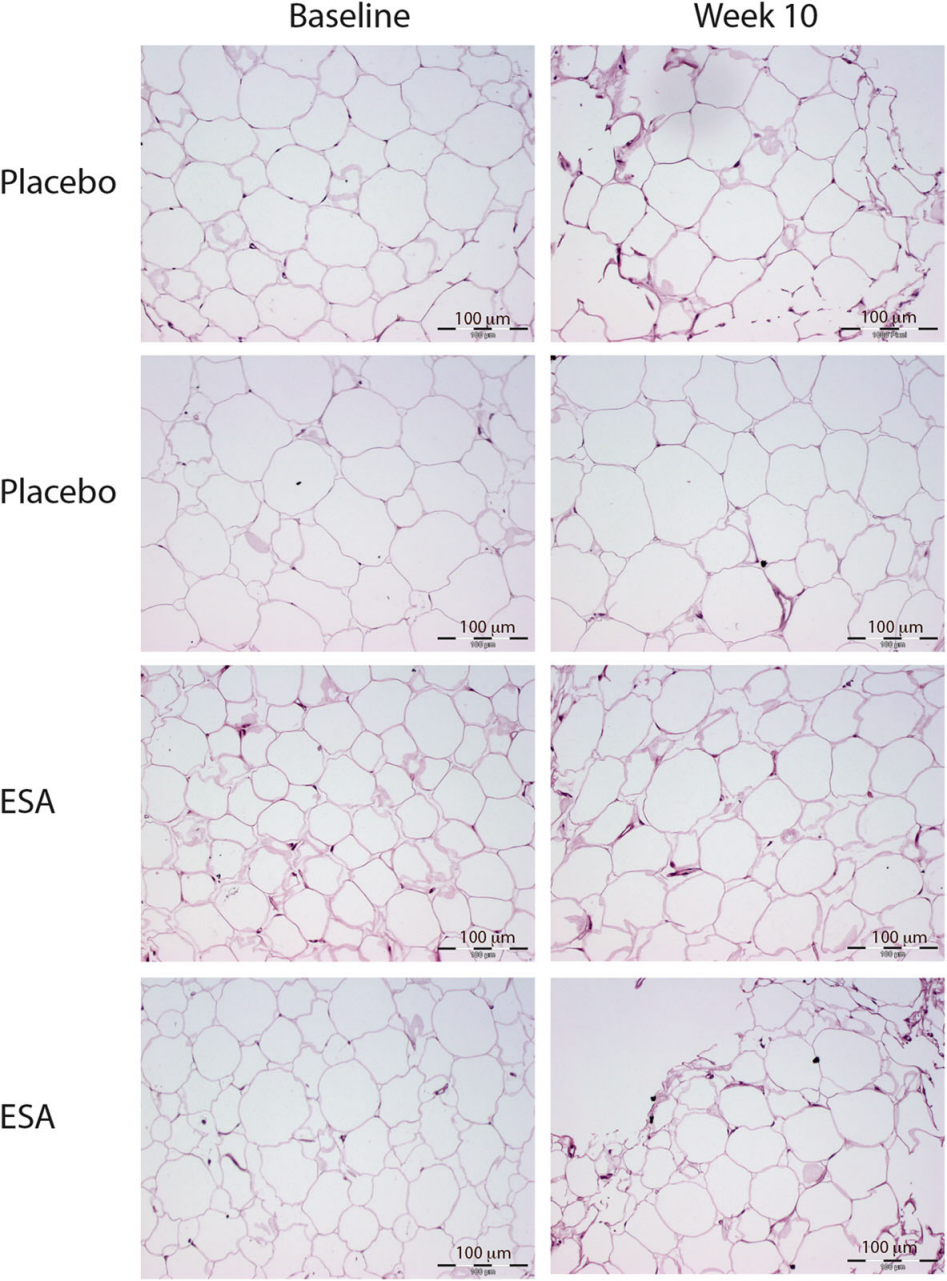
Haematoxylin/Eosin staining of WAT after prolonged ESA treatment. Haematoxylin/eosin staining of selected WAT samples from 2 subjects treated with placebo and 2 with ESA for 10 weeks

Selected WAT sections after prolonged treatment with ESA were stained with haematoxylin/Eosin in order to evaluate WAT morphology and visually evaluate adipocyte size. Biopsies from 2 subjects in each group were evaluated (Fig. 6), the morphology appeared normal and adipocyte size did not change substantially during the 10 weeks of placebo/ESA treatment.

### Alterations in mitochondrial proteins from WAT after prolonged ESA treatment

**Fig. 7:**
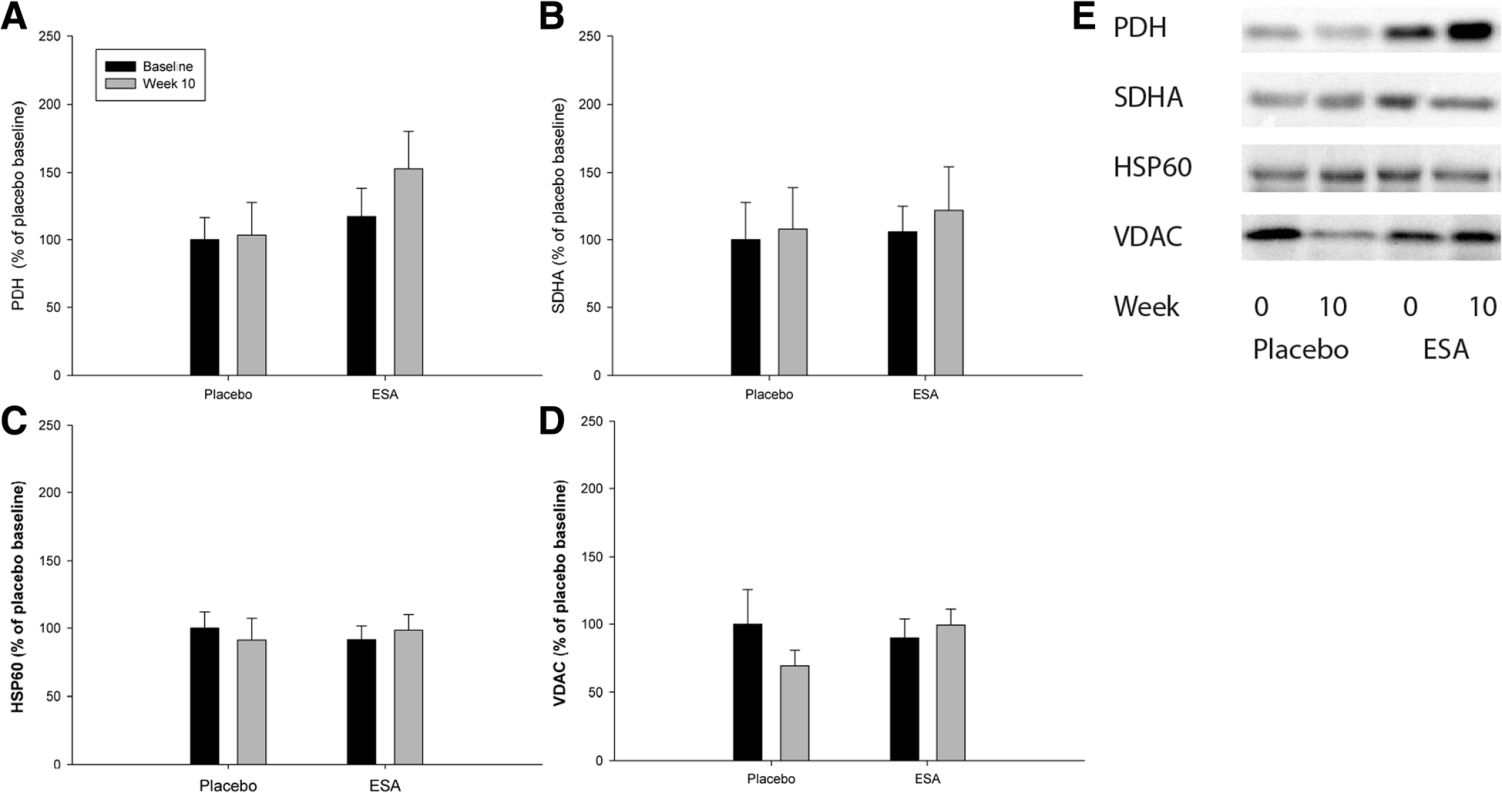
Mitochondrial protein levels after prolonged ESA treatment. Westernblot analysis of selected mitochondrial proteins in adipose tissue. Biopsies were collected before (*black bars*) and after 10 weeks (*gray bars*) of treatment with either placebo or ESA. Data were normalized to total protein level in the gel. Furthermore, the relative increases were measured and the placebo-baseline measurements were set to average 100 %. No effect of ESA treatment or time was found for any of the measured proteins (*n* = 9)

Different mitochondrial proteins (VDAC, HSP90, PDH, and SDHA) were measured before and after prolonged ESA treatment in order to evaluate mitochondrial protein content, as an indirect measure of mitochondrial biogenesis. None of these proteins changed in response to prolonged ESA treatment (Fig. 7). The level of uncoupling protein 1 (UCP1) was also measured as a marker of BAT in the human subcutaneous adipose biopsies. A band corresponding to UCP1 was detected in rat BAT but not in our human WAT biopsies (data not shown).

## Discussion

Previous studies in mice have shown significant effects of Epo treatment on adipose tissue lipolysis and total body weight. In human studies, FFA levels is increased both after acute and prolonged rHuEpo/ESA treatment [6, 7]. However, the current study did not confirm either acute or prolonged effects of rHuEpo/ESA treatment on adipose tissue lipases and signaling pathways involved in the regulation of lipolysis.

### Epo-R mRNA and protein and activation of signaling pathways in WAT

Mouse WAT contains a sizable amount of Epo-R mRNA [2], and Epo treatment associates with phosphorylation of Akt [9]. Moreover, mice lacking the Epo-R in non-hematopoietic tissues exhibit significantly reduced phosphorylation levels of p38MAPK, ERK42/44, and PPARγ [2]. Furthermore, phosphorylation of ERK42/44, p38MAPK, and PPARγ in preadipocytes does not depend on Epo-R expression under normal culture conditions, but during adipocyte differentiation the expression of Epo-R increases in concomitance with increased phosphorylation of ERK42/44, p38MAPK, PPARγ, Akt, and STAT5 [2, 19]. In our human study, we detected higher Epo-R mRNA levels in WAT than in bone marrow and than previously found in human skeletal muscle [13]. However, we were not able to measure any Epo-R protein in subcutaneous WAT and no evidence of pertinent signaling cascades related to the Epo-R after acute Epo exposure. Species and dosing differences could be an explanation for these conflicting results.

### Direct and indirect effects of Epo on lipolysis

The direct effects of Epo on lipolysis in WAT have not previously been investigated. Adipose triglyceride lipase (ATGL) converts triglycerides (TG) to diacylglycerol, and hormone sensitive lipase (HSL) hydrolyses diacylglycerol to monoacylglycerol, in the process of converting TG to FFA [20]. HSL and ATGL were not affected by rHUEpo/ESA treatment in the current studies.

ATGL is primarily activated by protein-protein interaction with the co-activator comparative gene identification-58 (CGI-58) [21]. G_0_/G_1_ switch gene 2 (G0S2) is identified as an inhibitor of ATGL. Inhibition by G0S2 appears to be dominant to activation by CGI-58 [22, 23]. In the current study, prolonged ESA treatment did not affect protein levels of CGI-58., nor did G0S2 change after acute or prolonged rHuEpo/ESA treatment.

Different lipid droplet proteins are involved in the regulation of lipolysis. Perilipin (PLIN1) regulates the activity of ATGL and HSL and their access to lipid substrates in the lipid droplet. Cidea and Cidec are negative regulators of lipolysis and promote lipid droplet stabilization and inhibit lipolysis by providing a barrier around the lipid core [20]. No alteration in PLIN1 or Cidea/Cidec was found in the current study.

Acetyl CoA Carboxylase (ACC) is involved in the regulation of beta-oxidation. In its active state ACC catalyzes the conversion of acetyl-CoA to malonyl-CoA, which inhibits beta-oxidation. Phosphorylation levels of ACC were not altered in the current studies. Insulin also affects lipolysis, with increased insulin levels inhibiting lipolysis and stimulating lipogenesis. Insulin levels were not altered in response to either acute or prolonged rHuEpo/ESA treatment [6, 7]. In addition, ESA treatment did not result in a visual reduction in adipocyte morphology evaluated from haematoxylin/eosin staining. It cannot be excluded that Epo can affect lipolysis in other indirect ways not evaluated in the current study.

### Epo and metabolic activity in white adipose tissue

High-dose Epo treatment to mice leads to pronounced reductions in body weight and knock-down of the Epo-R in non-hematopoietic tissues leads to dramatic elevations in WAT amount [2, 9]. In contradiction, specific knock-down of the Epo-R in mice adipose tissue did not affect either body weight or body composition [15]. In support hereof, in the current prolonged human study we did not observe any changes in weight or body composition [7].

Recently it was shown that Epo treatment leads to increased AMPKα phosphorylation both in preadipocytes and in WAT from diet-induced obese mice [24]. The increased AMPKα activity changed the NAD+/NADH ratio resulting in activation of Sirt1 followed by increased deacetylation of PGC-1α. Thus, Epo could through activation of the AMPKα-Sirt1-PGC-1α pathway acts as an energy sensor and modulate cellular redox state and energy homeostasis in adipocytes [24]. In the current study, AMPKα phosphorylation was not altered 1-h post rHuEpo administration.

Alterations in body weight and the amount of WAT could also be due to alterations in energy expenditure. In a mouse model, Epo was shown to affected activity levels, total oxygen consumption, and respiratory quotient (RQ) [2]. The authors speculated that Epo could directly stimulate the proopiomelanocortin (POMC) neurons in the hypothalamus to induce α-melanocyt-stimulating hormone production that exerts direct effects on food intake and energy homeostasis [2]. In support of this, adipose tissue specific deletion of Epo-R in mice did not significantly alter whole-body energy expenditure or RQ values [15]. Resting energy expenditure significantly increased in the current studies, and fat oxidation tended to be increased in relation to rHuEpo treatment [6, 7]. The effect of Epo on POMC was not measured in our human studies, hence, we cannot conclude on the neurological effects.

### Epo and mitochondrial content in white adipose tissue

Diet-induced obese mice treated with EPO exhibit increased CytC, Cpt1, and PGC-1α mRNA levels indicating an increased mitochondrial biogenesis [9]. In addition, Epo stimulation increases citrate synthase activity and fatty acid oxidation in both adipocytes and WAT from diet-induced obese mice [9]. Thus, Epo seems to increase cellular mitochondrial respiration and oxidative metabolism beyond its effects on increased oxygen transport in these animal studies. However, prolonged ESA treatment to humans did not support the hypothesis that ESA should induce alterations in mitochondrial content in humans.

In contradiction to WAT, BAT does not respond to Epo treatment in mice models [9]. However, Epo treatment seems to promote a BAT-like phenotype in WAT in mice with increased gene expression of e.g. Cidea, UCP1, UCP3, PPARα, and PGC-1α, resulting in mitochondrial biogenesis and increased uncoupled respiration [9]. In the current study, UCP1, Cidea and Cidec protein levels were not significantly altered after 10 weeks of ESA treatment, thus, ESA treatment does not seem to induce browning of WAT in humans.

## Conclusion

In contrast to alluring data in mice model, the present human studies do not support direct effects of Epo on adipose tissue lipases and signaling pathways involved in the regulation of lipolysis. Although Epo-R mRNA was detected in human WAT samples, this did not translate into detectable Epo-R protein, which is compatible with the absence of evidence of Epo-R signaling. However, it has to be acknowledged that the sample size in the current study is small and the variation substantial. It remains to be tested whether prolonged administration of Epo in supra-physiological amounts to human subjects may affect adipose tissue similar to what has been observed in animal studies.
